# Species of *Adialytus* Förster, 1862 (Hymenoptera, Braconidae, Aphidiinae) in Iran: taxonomic notes and tritrophic associations

**DOI:** 10.3897/zookeys.221.3541

**Published:** 2012-09-13

**Authors:** Ehsan Rakhshani, Petr Starý, Željko Tomanović

**Affiliations:** 1 Department of Plant Protection, College of Agriculture, University of Zabol, 98615-538, I .R. Iran; 2 Laboratory of Aphidology, Institute of Entomology, Biology Centre, Academy of Sciences of the Czech Republic, Branišovská 31, 37005, České Budějovicé, Czech Republic; 3 Institute of Zoology, Faculty of Biology, University of Belgrade, Studentski trg 16, 11000, Belgrade, Serbia

**Keywords:** *Adialytus*, taxonomy, host aphid associations, species complex

## Abstract

The species of *Adialytus* Förster in Iran are taxonomically studied and new data on distribution and host associations are presented. The existence of a species complex, in the case of *Adialytus ambiguus* (Haliday), and the morphological variability in commonly used taxonomic characters has been discussed. In total, four valid species belonging to the genus *Adialytus* including *Adialytus ambiguus* (Haliday), *Adialytus salicaphis* (Fitch), *Adialytus thelaxis* (Starý) and *Adialytus veronicaecola* (Starý) have been identified and recorded from Iran. Also, we recognized two additional phenotypes: “*Adialytus arvicola*” (Starý) and “*Adialytus* cf. *ambiguus*” (Haliday). These phenotypes and *Adialytus veronicaecola* are newly recorded from Iran in association with *Sipha* and *Aphis* species, respectively. An illustrated key for identification of the species and two variable phenotypes is presented.

## Introduction

The genus *Adialytus* Förster is morphologically very close to the genus *Lysiphlebus* Förster from which it can be differentiated by the absence of M & m-cu and r-m veins in the fore wing. It was classified as a subgenus of *Lysiphlebus* (Starý 1975, 1976, 1979), after validation by Mackauer and Starý (1967) and Mackauer (1968). Later, the generic status of *Adialytus* was also suggested by Shujauddin (1978) and supported in some phylogenetic analyses (Kambhampati et al. 2000). This genus includes a few species with Holarctic distribution extending from the Far East (Starý and Schlinger 1967, Takada 1968, 1979, Shujauddin 1978) to central Asia (Starý 1979), Europe (Kavallieratos et al. 2001, 2004, Starý 2006) and North America (Pike et al. 2000). Until now, seven valid species have been recognized within this genus, including *Adialytus salicaphis* (Fitch), *Adialytus thelaxis* (Starý), *Adialytus ambiguus* (Haliday), *Adialytus balticus* Starý & Rakauskas, *Adialytus veronicaecola* (Starý), *Adialytus kaszabi* Takada and *Adialytus fuscicornis* (Ashmead). The first three species have already been recorded from Iran (Starý et al. 2000, Rakhshani et al. 2007), and they are restricted to Chaitophorinae and Thelaxinae aphid hosts (Mackauer 1965, Starý 1975). Remaining species are associated with different aphids out of these groups (Starý and Rakauskas 1979, Starý and Juchnevič 1978, Pike et al. 2000).

There was considerable ambiguity about *Lysiphlebus confusus* Tremblay & Eady and *Adialytus ambiguus*. The first species name was selected by Tremblay and Eady (1978) for the material from Haliday’s collection that was incorrectly named *Lysiphlebus ambiguus* and described by Mackauer (1960). They also synonymized *Lysiphlebus (Adialytus) arvicola* Starý with *Lysiphlebus (Adialytus) ambiguus*. The synonymy has been followed by different authors (Mescheloff and Rosen 1990, Starý 1979).

Here we review the species of *Adialytus* in Iran, together with new data on their host associations and distribution. In addition, the possible existence of species complexes and morphological variability within genus are discussed.

## Material and methods

Samples of different host plants including wild and cultivated trees, shrubs and herbs bearing the aphid colonies were gently cut off and placed inside the semi-transparent plastic boxes. The collected material were subsequently transferred to the laboratory and kept under controlled conditions with temperature range of 24–28°C and RH: 60±5%, for 2-3 weeks until the emergence of the adult parasitoids. The rearing boxes were inspected daily to prevent the activity of emerging hyperparasitoids. Once detected, they were immediately removed from the rearing boxes. The emerged parasitoids were also carefully collected using an aspirator and dropped into 75% ethanol for further examination. A few specimens from each sample were carefully dissected and mounted in slides using a Hoyer medium. The ratio measurements were based on these slide-mounted specimens using an ocular micrometer. Additional material from European and central Asian countries were also used for comparison of the morphological variation. The characters of flagellar segments, clypeus, fore wing, first metasomal tergit (=petiole) and female genitalia were used for comparison and differentiation of the species, as well as to find the reliable characters for identification key. The external morphology was studied using a NIKON Eclips E200 microscope equipped with a SONY DSC digital camera.

**Figure 1.**
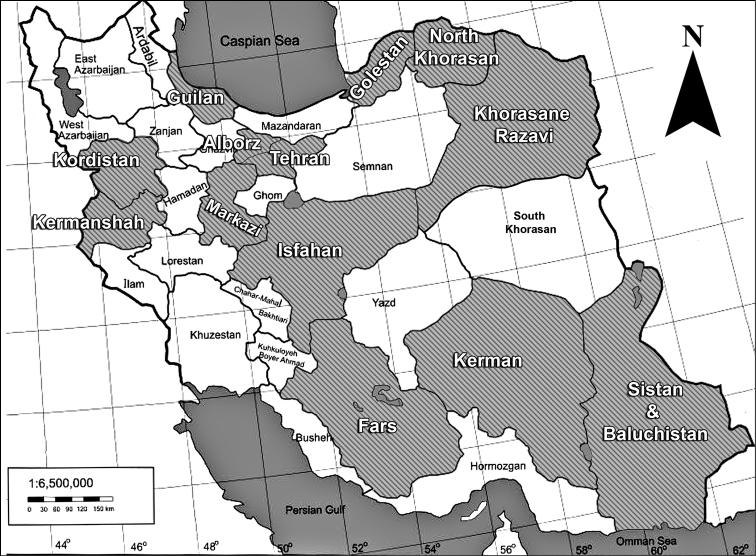
Map of the sampling areas at various parts of Iran, indicating 13 provinces.

The morphological terminology for parasitoids used in this paper follows Sharkey and Wharton (1997) and for the aphids Remaudière and Remaudière (1997), respectively. The nomenclature of host plants was based on Flora of Iran (Ghahreman 1978–2006). The specimens were deposited in the collection of the first author. Abbreviations of the names of provinces (Fig 1) are as follows: AL: Alborz, FA: Fars, GL: Golestan, GN: GUILAN, IS: Isfahan, KD: Kordiatan, KE: Kermanshah, KN: Kerman, KR: Khorasane Razavi, MA: Markazi, NK: North Khorasan, SB: Sistan & Baluchistan, TH: Tehran.

## Results

Four valid species of the genus *Adialytus*, as well as two additional phenotypes: “*Adialytus arvicola*” (Starý) and “*Adialytus* cf. *ambiguus*” (Haliday) (Table 1) were collected and identified in association with 14 aphid species on 15 host plants. Many specimens of *Adialytus ambiguus* (Haliday) were inconsistently different from examined specimens which originated in other countries. We categorized these specimens as “*Adialytus* cf. *ambiguus*”. *Adialytus veronicaecola* (Starý) and “*Adialytus arvicola*”(Starý) arenewly recorded from Iran. The latter species was reared from *Sipha* aphids which were also the specific hosts for *Adialytus ambiguus*. We found significant differences between the *Adialytus ambiguus* and “*Adialytus arvicola*” phenotype, based on the characters of fore wing, flagellar segments, hind legs, petiole (Table 2) and coloration. Additionally, a comparison with type specimens of *Adialytus arvicola* from the Czech Republic (Starý 1961a) clearly confirmed the existence of strong differences.

**Table 1.** A list of aphid-parasitoid associations.

**Aphid family**	**Aphid species**	**Parasitoid species**
Chaitophorinae	*Sipha maydis* Passerini	*Adialytus* cf. *ambiguus* (Haliday)*Adialytus arvicola* (Starý)
	*Sipha elegans* del Guercio	*Adialytus ambiguus* (Haliday)*Adialytus* cf. *ambiguus* (Haliday)*Adialytus arvicola* (Starý)
	*Sipha flava* (Forbes)	*Adialytus arvicola* (Starý)
	*Chaitophorus* spp.	*Adialytus salicaphis* (Fitch)
Thelaxinae	*Thelaxes suberi* (del Guercio)	*Adialytus thelaxis* (Starý)
Aphidiinae	*Aphis craccivora* Koch	*Adialytus veronicaecola* (Starý)<br/>
	*Aphis gossypii* Glover	
	*Aphis* sp.	

**Table 2.** The morphometric and meristic data for different characters of *Adialytus* species (Female) in Iran.

	F1^†^ *l/w*^‡^	F2 *l/w*	F3 *l/w*	F4 *l/w*	F1/F2 *length*	F1/F3 *length*	F1/F4 *length*	F1LP^§^	F2LP	Pt^#^ *l/w*	R1*^§§^*/Pt *length*	Setae on Clypeus	Petiole *l/w*	Ovipo-sitor sheath*l/w*
*Adialytus ambiguus*	2.10–2.30	2.40–2.60	1.90–2.10	1.80–2.00	1.00–1.10	0.90–1.00	0.90–1.00	0	1–2	2.90–3.00	1.30–1.40	4–5	1.80–2.00	2.90–3.20
*Adialytus* cf. *ambiguus*	2.60–2.85	2.70–2.90	2.70–2.85	1.80–2.00	0.90–1.00	0.90–1.10	0.90–1.10	0–1	2–3	2.85–3.10	0.90–1.10	4–5	1.80–2.00	2.80–3.20
*Adialytus arvicola*	2.50–2.80	2.10–2.45	2.20–2.40	1.70–1.90	0.90–1.10	0.90–1.10	0.95–1.20	0–1	2–4	3.00–3.20	0.70–0.80	6–8	2.00–2.20	2.80–3.10
*Adialytus salicaphis*	2.70–2.90	2.60–2.90	2.50–2.80	2.30–2.50	1.00–1.20	0.90–1.10	1.00–1.20	3–5	3–5	3.25–3.35	0.90–1.00	8–10	2.20–2.40	2.40–2.50
*Adialytus thelaxis*	1.60–1.70	1.50–1.60	1.50–1.60	1.60–1.70	1.00–1.20	1.00–1.20	0.90–1.10	3–5	4–6	2.80–3.10	0.90–1.00	8–10	1.80–2.00	2.60–2.70
*Adialytus veronicaecola*	2.00–2.20	1.90–2.00	1.90–2.00	2.05–2.15	1.00–1.10	1.00–1.10	1.00–1.10	0–1	0–1	3.00–3.20	0.60–0.70	6–8	1.90–2.20	2.15–2.30

^†^: F1–F4: Flagellomers 1–4^‡^ *l/w*: Length/width ratio^§^ LP: Longitudinal placodes*^§§^* R1: Radial vein 1 (= metacarpus)^#^: Pterostigma

### Key to *Adialytus* species in Iran (based on adult females)

**Table:** 

1	Ovipositor sheath considerably elongated, lengh/width ratio of 2.80–3.20 (Figs 6A–C)	2
–	Ovipositor sheath stout, length/width ratio of 2.20–2.70 (Figs 6D–F)	4
2	Vein R1 (= metacarpus) of fore wing 0.7–0.8 × as long as pterostigma (Fig 3C)	“*Adialytus arvicola*” (Starý)
–	Vein R1 of fore wing subequal (Fig 3B) or considerably longer (Fig 3A) than pterostigma	3
3	Vein R1 of fore wing 1.3–1.4 × as long as pterostigma, reaching apex of wing (Fig 3A)	*Adialytus ambiguus* (Haliday)
–	Vein R1 of fore wing 0.9–1.1 × as long as pterostigma, not reaching apex of wing (Fig 3B)	*“Adialytus* cf. *ambiguus”* (Haliday)
4	Flagellar segments (Fig 2E) subquadrate, slightly longer than their maximum width, *l/w* ratio of 1.5–1.6. Flagellar segments (Fig 2E) and hind femur (Fig. 4E) covered with long and prevalently erect setae. Ovipositor sheath sharply angular (Fig 6E)	*Adialytus thelaxis* (Starý)
–	Flagellar segments (Figs 2D, 2F) cylindrical, considerably longer than their maximum width, *l/w* ratio of 2.0–2.9. Flagellar segments and hind femur covered with adpressed (Figs 2F, 4F) or semi-erect (Fig 2D, 4D) setae. Ovipositor sheath roundly angular (Figs 6D, 6F)	5
5	First metasomal tergite (petiole) elongate, 2.2–2.4 × as long as wide at level of spiracles (Fig 5D). Flagellar segments covered with prevalently semi-erect setae which are equal to diameter of segment. Flagellomere 1 bearing 3–4 longitudinal placodes (Fig 2D). Hind femur covered with prevalently semi-erected setae (Fig 4D)	*Adialytus salicaphis* (Fitch)
–	First metasomal tergite (petiole) short, 1.9–2.1X as long as wide at spiracles (Fig 5F). Flagellar segments covered with adpressed setae which are distinctly shorter than diameter of segment. Flagellomere 1 with 0–1 longitudinal placode (Fig 2F). Hind femur covered with short adpressed setae (Fig. 4F)	*Adialytus veronicaecola* (Starý)

### List of *Adialytus* species and their host associations

#### Adialytus ambiguus

(Haliday, 1834)

http://species-id.net/wiki/Adialytus_ambiguus

Figs 2A 3A 4A 5A 6A

Aphidius ambiguus Haliday, 1834: 104–105.

##### Material examined.

1♂ 1‌♀, *Sipha elegans* del Guercio on *Triticum aestivum*, FA, Shiraz (29°34'22"N, 52°41'58"E, 1489 m), 27.IV.2005, 1♂ 1‌♀, coll.: E. Rakhshani.

##### Comments:

This species is closely related to other parasitoids of *Sipha* aphids, in its elongated ovipositor sheath (Fig 6A) and triangular shape of petiole which bears anterior and spiracular tubercles (Fig 5A). It can be differentiated from other species in having an extremely long vein R1 (= metacarpus) (Fig 3A). The hind femur and tibia are covered with both short and prevalently erect long setae (Fig. 4A).

#### Adialytus ambiguus

(Haliday, 1834) cf.

Figs 2B 3B 4B 5B 6B

##### Material examined.

22♂ 20♀,*Sipha maydis* Passerini on *Bromus tectorum*, NK, Gharemeidan (37°25'42"N, 56°33'19"E, 1544 m), 14.V.2008, 15♂ 18♀, coll. S. Kazemzadeh; *Sipha elegans*del Guercio on *Gastridium phleoides*, IS, Nazhvan (32°38'25"N, 51°35'48"E, 1582 m), 05.IX.2011, 7♂ 2‌♀, coll. E. Nader.

##### Comments.

**Figure 2.**
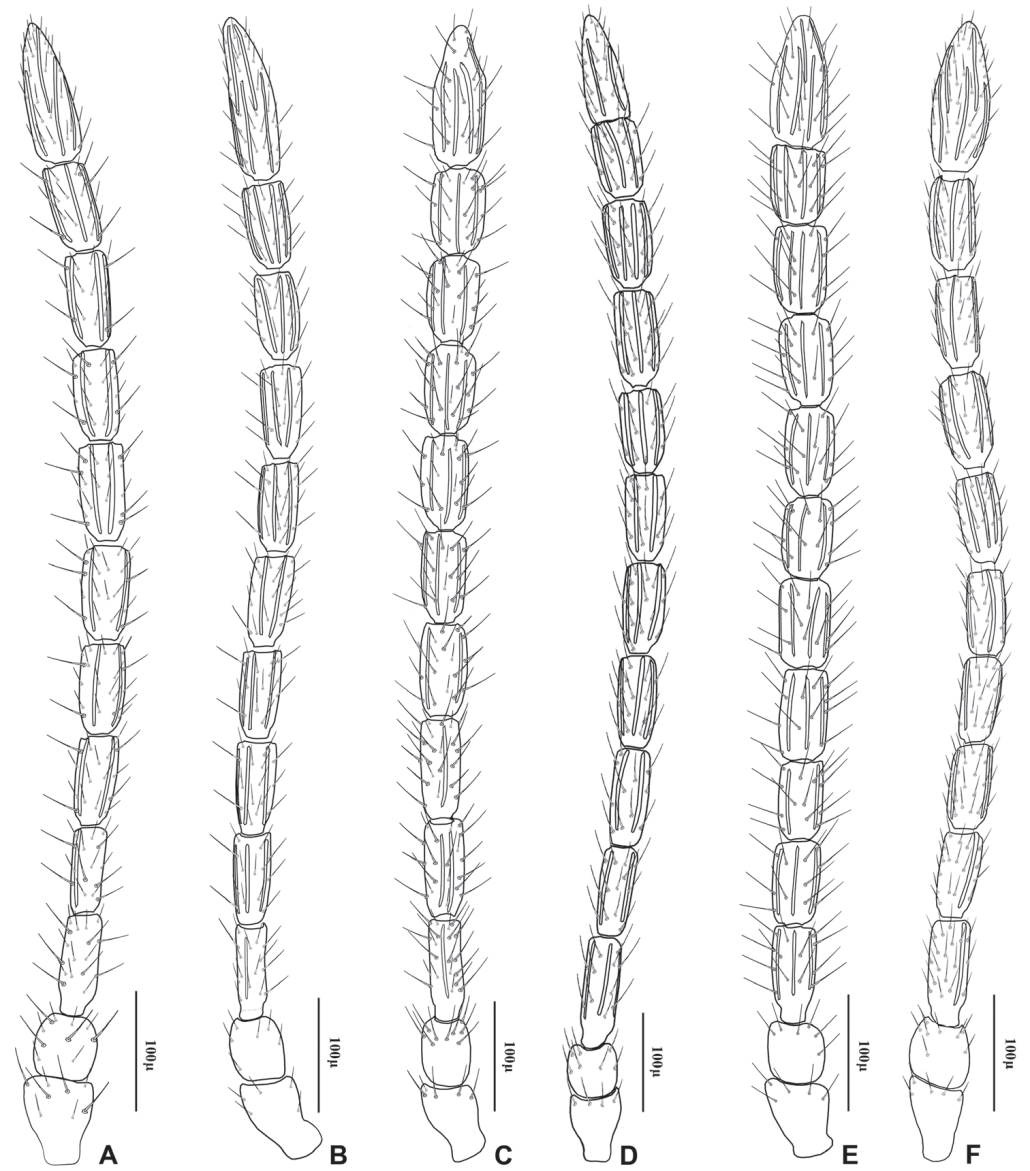
Antenna of *Adialytus* species **A** *Adialytus ambiguus* **B** *Adialytus* cf. *ambiguus* **C** *Adialytus arvicola* **D** *Adialytus salicaphis* **E** *Adialytus thelaxis* **F** *Adialytus veronicaecola*.

**Figure 3.**
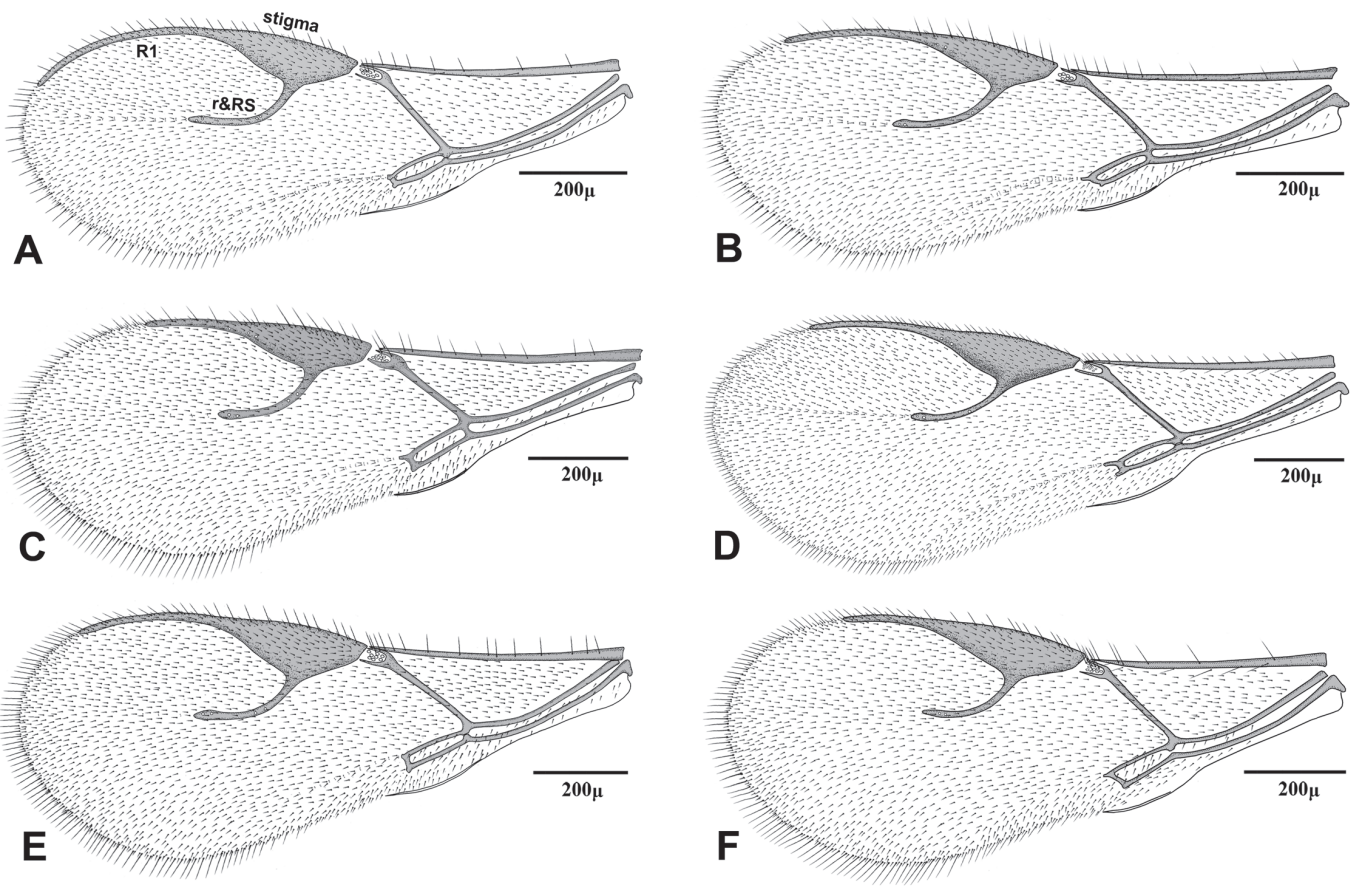
Fore wing of *Adialytus* species **A** *Adialytus ambiguus* **B** *Adialytus* cf. *ambiguus* **C** *Adialytus arvicola* **D** *Adialytus salicaphis* **E** *Adialytus thelaxis* **F** *Adialytus veronicaecola*.

**Figure 4.**
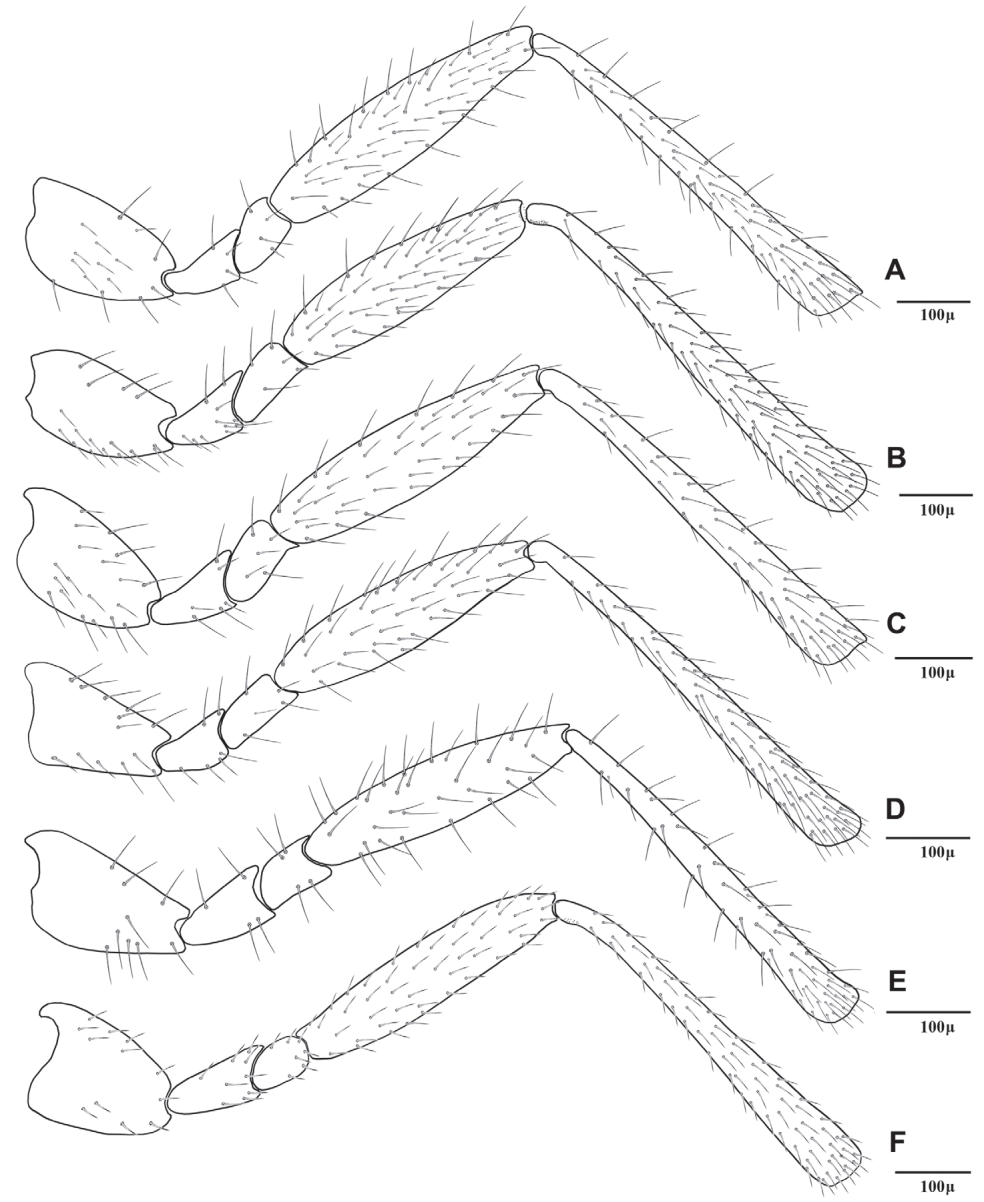
Hind leg of *Adialytus* species, excluding tarsomeres **A** *Adialytus ambiguus* **B** *Adialytus* cf. *ambiguus* **C** *Adialytus arvicola* **D** *Adialytus salicaphis* **E** *Adialytus thelaxis* **F** *Adialytus veronicaecola*.

**Figure 5.**
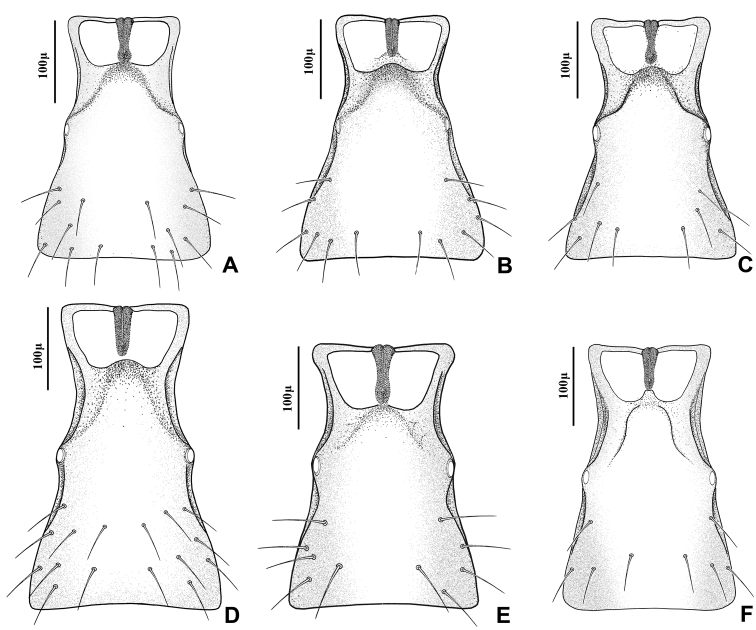
Petiole or first metasomal tergite of *Adialytus* species **A** *Adialytus ambiguus* **B** *Adialytus* cf. *ambiguus* **C** *Adialytus arvicola* **D** *Adialytus salicaphis* **E** *Adialytus thelaxis* **F** *Adialytus veronicaecola*.

**Figure 6.**
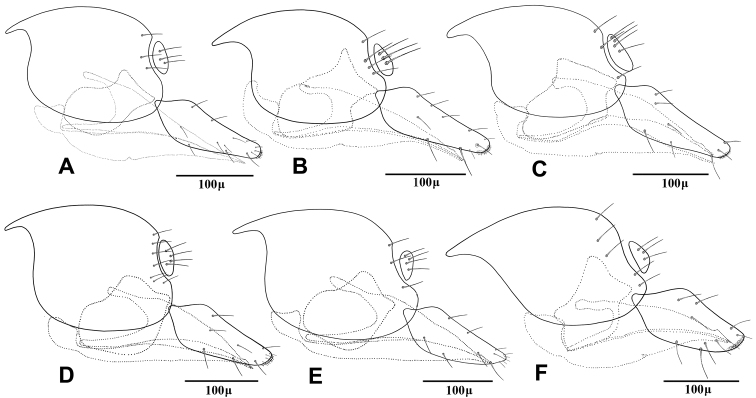
Female genitalia of *Adialytus* species **A** *Adialytus ambiguus* **B** *Adialytus* cf. *ambiguus* **C** *Adialytus arvicola* **D** *Adialytus salicaphis* **E** *Adialytus thelaxis* **F** *Adialytus veronicaecola*.

The specimens normally run to *Adialytus ambiguus* according to the general characters of the first metasomal tergite (Fig 5B), ovipositor sheath (Fig 6B), the flagellomeres (Fig 2B) and the setae on the hind femur (Fig 4B). It can be differentiated from *Adialytus ambiguus* by having the shorter vein R1 that is 0.9–1.1 × as long as pterostigma that does not reach the apex of the fore wing (Fig 3B). It can be separated from *Adialytus arvicola* (Fig 3C), by its longer vein R1.

#### Adialytus arvicola

(Starý, 1961)

http://species-id.net/wiki/Adialytus_arvicola

Figs 2C 3C 4C 5C 6C

Lysiphlebus arvicola Starý, 1961a: 98–100.

##### Material examined.

38♂ 63♀, *Sipha flava*(Forbes) on ‌*Agropyrum repens*, KE, Kermanshah (34°19'33"N, 47°05'53"E, 1322 m), 25.VI.2011, 22‌♂ 55‌♀, coll. Y. Nazari; *Sipha maydis*Passerini on *Avena fatua*, KE, Kermanshah (34°19'33"N, 47°05'53"E, 1322 m), 11.VI.2011, 2♂, coll. Y. Nazari; on *Bromus tectorum*, KE, Sanandaj (35°17'52"N, 46°59'59"E, 1517 m), 16.V.2005, 1♂, coll. E. Rakhshani; on *Cynodon dactylon*, KN, Kerman (30°14'28"N, 57°07'20"E, 1775 m), 22.XI.2007, 6♂ 2♀, coll. H. Barahoei; on *Sorghum halepense*, KE, Kermanshah (34°19'35"N, 47°06'00"E, 1320 m), 11.VI.2011, 2‌♂ 3‌♀, coll.: Y. Nazari; *Sipha elegans* del Guercio on *Triticum aestivum*, KR, Mashhad (36°15'22"N, 59°28'42"E, 1164m), 12.IV.2012, 5♂ 3♀, coll. J. Karimi.

##### Comments.

Generally this species can be confused with other *Adialytus* species on *Sipha* aphids, but it is immediately distinguishable by its very short vein R1 (0.7–0.8 × as long as pterostigma) (Fig 3C). Also, its petiole has much stronger anterior and spiracular tubercles (Fig 5C). Most of the metasoma is yellowish, while in other *Adialytus* species it is uniformly brown to dark brown.

#### Adialytus salicaphis

(Fitch, 1855)

http://species-id.net/wiki/Adialytus_salicaphis

Figs 2D 3D 4D 5D 6D

Trioxys salicaphis Fitch, 1855: 841.

##### Material examined.

138♂ 223‌♀, *Chaitophorus euphraticus* Hodjat on *Populus euphratica*, SB, Zahedan (29°23'27"N, 60°48'49"E, 1498 m), 24.III.2003, 3♂ 7‌♀, coll. E. Rakhshani; *Chaitophorus remaudierei* Pintera on *Salix alba*, KD, Marivan (35°31'33"N, 46°09'21"E, 1293 m), 08.X.2004, 4♂ 6‌♀, coll. E. Rakhshani; *Chaitophorus salijaponicus niger* Mordvilko on *Salix alba*, FA, Sepidan (30°15'55"N, 51°58'43"E, 2244 m), 23.V.2009, 7♂ 9‌♀, coll. S. Taheri; NK, Shirvan, 24.VI.2008, 32♂ 54♀, coll. S. Kazemzadeh; NK, Esfarayen (37°05'12"N, 57°30'39"E, 1293 m), 17.V.2008, 8♂ 13♀, coll. S. Kazemzadeh; *Chaitophorus populialbae* (Boyer de Fonscolombe) on *Populus alba*, AL, Karadj (35°44'45"N, 51°10'07"E, 1296 m), 09.X.2002, 16♂ 29♀, coll. E. Rakhshani; *Chaitophorus populeti*(Panzer) on *Populus nigra*, TH, Tehran (35°47'52"N, 51°24'08"E, 1650 m), 09.XI.2002; 32♂ 48♀ coll. E. Rakhshani; *Chaitophorus leucomelas*Koch on *Populus nigra*, KN, Lalezar (29°31'05"N, 56°48'59"E, 2845 m), 09.X.2007, 5♂ 15♀, coll. H. Barahoei; AL, Karadj (35°55'06"N, 51°05'04"E, 1875 m) 27.VI.2003; 11♂ 18♀, coll. E. Rakhshani; on *Populus* sp. FA, Sepidan (30°15'55"N, 51°58'43"E, 2244 m), 22.V.2009, 8♂ 12♀, coll.: S. Taheri; *Chaitophorus vitellinae*(Schrank) on *Salix alba*, MA, Mahallat (33°53'12"N, 50°27'31"E, 1652 m), 22.IV.2005, 5♂ 4♀, coll.: E. Rakhshani; *Chaitophorus*sp., on *Populus alba*, NK, Shirvan (37°23'35"N, 57°54'40"E, 1082 m), 24.V.2008, 7♂ 8♀, coll. S. Kazemzadeh.

##### Comments.

*Adialytus salicaphis* differs from other congeners in having very elongated first metasomal tergite (petiole) (Fig 5D), and short and dense marginal setae of the fore wing (Fig 3D). It can also be differentiated from *Adialytus arvicola* by the number of longitudinal placodes on flagellomere 1 (3–5 in *Adialytus salicaphis* vs. 0–1 in *Adialytus arvicola*). The specimens of *Adialytus salicaphis* associated with *Salix* spp., especially those reared from *Chaitophorus salijaponicus niger* on *Salix alba*, were slightly different from the specimens that reared from *Chaitophorus* spp. on *Populus*. The major differences were the lesser number of setae on the clypeus (4–5 vs. 8–10), lesser longitudinal placodes on the first flagellomere (1–2 vs. 3–5) and predominantly adpressed and short setae on the flagellomeres and hind femur compared with the long semi-erect to erect setae among the short setae in specimens from *Populus*.

#### Adialytus thelaxis

(Starý, 1961)

http://species-id.net/wiki/Adialytus_thelaxis

Figs 2E 3E 4E 5E 6E

Lysiphlebus thelaxis Starý, 1961a: 100–101.

##### Material examined.

11♂ 26♀, *Thelaxes suberi* (del Guercio) on *Quercus* sp., GN, Rasht (37°17'24"N, 49°35'43"E, -4 m), 24.V.2004, 4♂ 3♀, coll.: E. Rakhshani; on *Quercus castanifolia*, GL, Gorgan (36°47'33"N, 54°27'02"E, 340 m), 06.IV.2010, 7♂ 23♀, coll. A. Sargazi.

##### Comments.

This species can be easily separated from other congeners by having mainly erect long setae on the flagellomeres (Fig 2E) and the hind femur (Fig 4E). The setae on the postero-dorsal aspect of petiole are similar (Fig 5E). Additionally, *Adialytus thelaxis* is the only species with a sharply pointed ovipositor sheath (Fig 6E).

#### Adialytus veronicaecola

(Starý, 1978)

http://species-id.net/wiki/Adialytus_veronicaecola

Figs 2F 3F 4F 5F 6F

Lysiphlebus veronicaecola Starý, 1978: 528–529.

##### Material examined.

2♂ 3♀, *Aphis craccivora*Koch on *Phaseolus vulgaris*, IS, Flavarjan (32°30'56"N, 51°29'02"E, 1618 m), 2♀, coll. E. Nader; *Aphis* sp. on *Rubia tinctorum*, IS, Mobarakeh (32°30'56"N, 51°30'17"E, 1658 m), 13.XI.2010, 1♂ 1♀, coll. E. Nader; *Aphis gossypii*Glover on *Cucurbita pepo*,IS, Ghahderijan (32°36'18"N, 51°28'25"E, 1611 m), 05.XI.2010, 1♂, coll. E. Nader.

##### Comments.

This species is unique in that it was reared from *Aphis* species. According to the general characters of the fore wing (Fig 3F), petiole or first metasomal tergite (Fig 5F) and the ovipositor sheath (Fig 6F) it is closely related to *Adialytus salicaphis* from which it can be immediately distinguished in having prevalently short and adpressed setae on the flagellomeres (Fig 2F) and hind femur (Fig 4F). It can also be differentiated from *Adialytus salicaphis* by having lesser longitudinal placodes on flagellomeres 1 and 2 (0–1 in *Adialytus veronicaecola* vis 3–5 in *Adialytus salicaphis*). In addition, *Adialytus veronicaecola* differs from the other species in having a stout ovipositor sheath with a strongly convex postero-dorsal outline (Fig 6F).

## Discussion

In a biological aspect, the host range pattern of *Adialytus* species can be used as an appropriate criterion supporting its generic status as separate from, but closely related to the genus *Lysiphlebus* Förster. Species of the genus *Lysiphlebus* are mostly parasitoids of the genera *Aphis* and *Brachycaudus* (Starý 1999, 2006, Starý et al. 1998) but, exceptionally, include some other aphid groups such as *Metopeurum* (Macrosiphini) in the case of *Lysiphlebus hirticornis* Mackauer (Mackauer 1960, Starý 1961b). On the other hand, about half of the *Adialytus* speciesare associated with different aphid subfamilies consisting of Thelaxinae and Chaitophorinae, while others attack *Aphis* (Starý and Juchnevič 1978, Pike et al. 2000) and *Dysaphis* (Starý and Rakauskas 1979). It can be suggested here that the members of the latter group are biologically more closely related to the genus *Lysiphlebus*. The Nearctic species, *Adialytus fuscicornis* (Ashmead), a parasitoid of *Aphis* species (Pike et al. 2000) tends also to resemble morphologically the *Lysiphlebus* species except for its more reduced wing venation. Among the recorded species, *Adialytus veronicaecola* manifests two major diagnostic characters including the stout ovipositor sheath and prevalently adpressed setae on the flagellar segments and hind legs. Other species have a more elongated ovipositor sheath and different types of chaetotaxy bearing both semi-erect and erect setae. In contrast, *Adialytus balticus* Starý has erect and perpendicular setae on the flagellomeres. The habitat and host associations of this species on the root collar of *Anthriscus* sp. (Starý and Rakauskas 1979) might be the reason for having perpendicular setae on the flagellomeres as well as the reduction in length of the segments (Starý et al. 1998). So, we lack clear diagnostic characters for separation of these groups given the present state of knowledge.

*Adialytus veronicaecola* was originally described from Kazakhstan (Starý and Juchnevič 1978, Starý 1979). The new evidence also supports the original distribution of this species in central Asia, as well as host specificity on *Aphis* species. Three other *Aphis* species are added to the list of its host, of which *Adialytus craccivora* and *Adialytus gossypii* are of economic importance. “*Adialytus arvicola*”phenotype is also newly recorded from Iran, but the earlier records are most probably cited under the synonymy with *Adialytus ambiguus*. While it can be considered as the first evidence of the existence of a species complex in the case of *Adialytus ambiguus*, it sounds to be a rather specific parasitoid of *Sipha* aphids of various subgenera including *Atheroides* Haliday, *Chaetosiphoniella* and *Sipha* Passerini (Mackauer 1965), “*Adialytus arvicola*”seems to be restricted to the later subgenus (Starý 1961a, b). On the other hand, the separation of these two species, as well as the intermediate “*Adialytus* cf. *ambiguus*”, cannot be clearly justified without molecular analyses, since they were collected from almost the same host aphids at the studied area. Generally, *Adialytus ambiguus* seems to be a very rare species in Iran, and it might be replaced by the geographical species/subspecies manifesting significant morphological differences. The most important diagnostic character is in the pattern of the venation of the fore wing.

It is yet unclear which “phenotype” of *Adialytus ambiguus* was used for the phylogenetic analyses (Kambhampati et al. 2000, Sanchis et al. 2000) but, nominally, the genus *Adialytus* was classified as a paraphyletic group due to the arrangement of *Adialytus ambiguus* inside the genus *Lysiphlebus* (Sanchis et al. 2000). On the other hand, “*Adialytus arvicola*”was grouped with the other *Adialytus* species, separated from *Lysiphlebus* spp. (Kambhampati et al. 2000). Differences among the specimens of *Adialytus salicaphis* associated with *Salix* and *Populus* seem to be an intra-specific variation together with some other characters including the length/width ratio of petiole and carination of the propodeum (see Takada 1979). Shujauddin (1978) also found the same difference between the Indian and European specimens. These variations should be considered in further taxonomical studies.

## Conclusion

In general, identification of the *Adialytus* species merely based on the morphological characters is rather difficult, since they are very similar and even these characters may be contributed to intraspecific variation. Nevertheless, the host range patterns which are mostly specific can be greatly useful for separation of most species, excluding taxa in the *Adialytus ambiguu*sspecies complex, which have almost the same host range. Further investigations based on the geometric morphometric analysis, as well as suitable molecular markers might reveal the exact identity of the above-mentioned taxa and status “*Adialytus arvicola*” and “*Adialytus* cf. *ambiguus*”. Furthermore, a re-classification at a tribal level is necessary to reconstruct the relationships between two groups of *Adialytus* species and their position compared to the genus *Lysiphlebus*.

## Supplementary Material

XML Treatment for Adialytus ambiguus

XML Treatment for Adialytus ambiguus

XML Treatment for Adialytus arvicola

XML Treatment for Adialytus salicaphis

XML Treatment for Adialytus thelaxis

XML Treatment for Adialytus veronicaecola

## References

[B1] Fitch A (1855) First report on the noxious, beneficial and other insects of the state of New York made to the State Agricultural Society, persuant to approbation for this purpose from the Legislature of the State. Transactions of the New York State Agricultural Society 14: 705-880.

[B2] Haliday AH (1834) Essay on the classification of parasitic Hymenoptera of Britain, which correspond with the Ichneumones minuti of Linnaeus. Entomologist’s Monthly Magazine 2: 93–106.

[B3] Ghahreman A (1978–2006) Flora of Iran. Research Institute of Forest an Rangelands and Tehran University Press, Tehran, Vol. 1–25.

[B4] Kambhampati S Völkl W Mackauer M (2000) Phylogenetic relationship among genera of Aphidiinae (Hymenoptera: Braconidae) based on DNA sequence of the mitochondrial 16S rDNA gene. Systematic Entomology 25: 437-445. doi: 10.1046/j.1365-3113.2000.00129.x

[B5] Kavallieratos NG Lykouressis DP Sarlis GP Stathas GJ Sanchis Segovia A Athanassiou CG (2001) The Aphidiinae (Hymenoptera: Ichneumonoidea: Braconidae) of Greece. Phytoparasitica 29: 306-340. doi: 10.1007/BF02981847

[B6] Kavallieratos NG Tomanović Ž Starý P Athanassiou CG Sarlis GP Petrović O Niketić M Veroniki MA (2004) A survey of aphid parasitoids (Hymenoptera: Braconidae: Aphidiinae) of Southeastern Europe and their aphid-plant associations. Applied Entomology and Zoology 39: 527-563. doi: 10.1303/aez.2004.527

[B7] Mackauer M (1960) Die europäischen Arten der Gattung *Lysiphlebus* Förster. Beiträge zur Entomologie 10: 582-623.

[B8] Mackauer M (1965) Parasitological data as an aid in aphid classification. Canadian Entomologist 97: 1016-1024. doi: 10.4039/Ent971016-10

[B9] Mackauer M (1968) Hymenopterorum Catalogus. Part 3. Aphidiidae. Dr. W Junk, The Hague, 103 pp.

[B10] Mackauer M Starý P (1967) Hymenoptera, Ichneumonoidea, World Aphidiidae. In: Delucchi V Remaudière G (Eds) Index of Entomophagous Insects. Le Francois, Paris, 1–195.

[B11] Mescheloff E Rosen D (1990) Biosystematic studies on the Aphidiidae of Israel (Hymenoptera: Ichneumonoidea) 3. The genera *Adialytus* and *Lysiphlebus*. Israel Journal of Entomology 24: 35-50.

[B12] Pike KS Starý P Miller T Graf G Allison L Boydston L Miller R (2000) Aphid parasitoids (Hymenoptera: Braconidae: Aphidiinae) of northwest USA. Proceedings of the Entomological Society of Washington 102: 688-740.

[B13] Rakhshani E Talebi AA Starý P Tomanović Ž Manzari S (2007) Aphid parasitoid (Hymenoptera: Braconidae: Aphidiinae) associations on willows and poplars in the biocorridors of Iran. Acta Zoologica Academiae Scientiarum Hungaricae 53: 281-297.

[B14] Remaudière G Remaudière M (1997) Catalogue of the world’s Aphididae. INRA, Paris, 475 pp.

[B15] Sanchis A Latorre A Gonzalez-Candelas F Michelena JM (2000) An 18S rDNA-based molecular phylogeny of Aphidiinae (Hymenoptera: Braconidae). Molecular Phylogenetics and Evolution 14: 180-194. doi: 10.1006/mpev.1999.0701 10679154

[B16] Sharkey MJ Wharton RA (1997) Morphology and terminology. In: Wharton RA Marsh PM Sharkey MJ (Eds) Manual of the New World genera of the familyBraconidae (Hymenoptera). Special Publication 1, International Society of Hymenopterists, 19–37.

[B17] Shujauddin (1978) New record of *Adialytus* Förster (Hymenoptera: Aphidiidae) from India with comments on the validity of the genus. Journal of Entomological Research 2 (2): 160-162.

[B18] Starý P (1961a) Taxonomic notes on the genus *Lysiphlebus* Förster (Hymenoptera: Aphidiidae). Bulletin Entomologique de Pologne 31: 97-103.

[B19] Starý P (1961b) Faunistic survey of Czechoslovak species of the genera *Lysiphlebus* Förster and *Trioxys* Haliday. Acta Faunistica Entomologica Musei Nationalis Pragae 7: 131-149.

[B20] Starý P (1975) The subgeneric classification of *Lysiphlebus* Förster, 1862 (Hymenoptera, Aphidiidae). Annotationes Zoologicae et Botanicae, Bratislava 105: 1-9.

[B21] Starý P (1976) Aphid Parasites (Hymenoptera, Aphidiidae) of the Mediterranean Area. Transactions of the Czechoslovak Academy of Sciences, Series of Mathematical and Natural Sciences and Dr. W Junk, The Hague, 95 pp.

[B22] Starý P (1979) Aphid Parasites (Hymenoptera, Aphidiidae) of the Central Asian Area. Transactions of the Czechoslovak Academy of Sciences, Series of Mathematical and Natural Sciences, Dr. W Junk, The Hague, 116 pp.

[B23] Starý P (1999) Biology and distribution of microbe-associated thelytokous population of aphid parasitoids (Hym., Braconidae, Aphidiinae). Journal of Applied Entomology 123: 231-235. doi: 10.1046/j.1439-0418.1999.00345.x

[B24] Starý P (2006) Aphid Parasitoids of the Czech Republic (Hymenoptera: Braconidae: Aphidiinae). Academia, Praha, 430 pp.

[B25] Starý P Juchnevič LA (1978) Aphid parasites (Hymenoptera: Aphidiidae) from Kazakhstan, USSR. Bulletin Entomologique de Pologne 48: 523-532.

[B26] Starý P Rakauskas RP (1979) *Adialytus balticus* sp. n., a parasitoid of *Dysaphis anthrisci* from the East Baltic (Hymenoptera, Aphidiidae; Homoptera, Aphididae). Acta Entomologica Bohemoslovaca 76: 313-317.

[B27] Starý P Schlinger E (1967) A Revision of the Far East Asian Aphidiidae (Hymenoptera). Series. Entomologica, No. 3, Dr. W Junk, The Hague, 204 pp.

[B28] Starý P Remaudière G González D Shahrokhi S (2000) A review and host associations of aphid parasitoids (Hymenoptera: Braconidae: Aphidiinae) of Iran. Parasitica 56 (1): 15-41.

[B29] Starý P Tomanović Ž Petrović O (1998) A new parasitoid of root feeding aphids from the Balkan mountains (Hymenoptera: Braconidae: Aphidiinae). Deutsche Entomologiche Zeitschrift 45: 175-179.

[B30] Takada H (1968) Aphidiidae of Japan (Hymenoptera). Insecta Matsumurana 30: 67-124.

[B31] Takada H (1979) Aphidiidae (Hymenoptera) from Mongolia. Folia Entomologica Hungarica 32 (1): 189-202.

[B32] Tremblay E Eady RD (1978) *Lysiphlebus confusus* sp.n. for *Lysiphlebus ambiguus* sensu auct. nec Haliday (1834) (Hymenoptera Ichneumonoidea). Bollettino del Laboratorio di Entomologia Agraria ‘Filippo Silvestri’ 35: 180-184.

